# Explanation of the PLC Effect in Advanced High-Strength Medium-Mn Steels. A Review

**DOI:** 10.3390/ma12244175

**Published:** 2019-12-12

**Authors:** Aleksandra Kozłowska, Barbara Grzegorczyk, Mateusz Morawiec, Adam Grajcar

**Affiliations:** Department of Engineering Materials and Biomaterials, Silesian University of Technology, 18A Konarskiego Street, 44-100 Gliwice, Poland; aleksandra.kozlowska@polsl.pl (A.K.); barbara.grzegorczyk@polsl.pl (B.G.); mateusz.morawiec@polsl.pl (M.M.)

**Keywords:** medium-manganese steel, Portevin–Le Chatelier phenomenon, dynamic strain ageing, Transformation-Induced Plasticity, retained austenite, deformation temperature

## Abstract

The paper reviews the recent works concerning the Portevin–Le Chatelier (PLC) effect in Advanced High-Strength Steels (AHSSs) with a special attention to high-strength medium-manganese steels. Theories explaining the mechanism of the plastic instability phenomenon in steels with medium- and high-Mn contents were discussed. The relationships between microstructural effects such as TRIP (Transformation-Induced Plasticity), TWIP (Twinning-Induced Plasticity) and the PLC effect were characterized. The effects of processing conditions including a deformation state (hot-rolled and cold-rolled) and strain parameters (deformation temperature, strain rate) were addressed. Factors affecting the value of critical strain for the activation of serrated flow behavior in particular in medium-manganese steels were described.

## 1. Introduction

The plastic instability phenomenon was firstly described by Albert Portevin and Francois Le Chatelier in 1923. They observed characteristic oscillations on strain–stress curves in aluminum-based alloys and low-carbon steels [1]. Since then, the Portevin–Le Chatelier effect (PLC) has been extensively studied in copper and aluminum-based alloys during tensile or compression tests [2,3,4,5]. However, in the case of steels the PLC effect was studied rarely [5,6,7]. In available literature, there are several theories explaining the mechanism of serrated flow in steels. Generally, a value of critical strain for activation of the PLC effect is related to several factors, which can be classified as interior factors: solute atom concentration (especially C and N content), dislocation density, grain size and external factors, including deformation temperature and strain rate [3,4,8,9]. The heterogeneous deformation associated with the increase in flow stress can lead to multiple cracks during sheet molding. In addition, delayed cracking may occur after the deep drawing process is completed. Temperature and strain rate factors affecting the PLC phenomenon deserve a special attention because of a technological point of view. During forming operations of sheets performed at various deformation rates, some amount of heat is generated, which affects the appearance and intensity of the plastic instability phenomenon [10].

Medium-manganese steels (3–12% wt % Mn) showing a Transformation-Induced Plasticity (TRIP) effect have attracted a significant attention in the automotive, thanks to their advantageous strength–ductility balance [11,12]. However, industrial application of those steels for body-in-white automobile components depends on their formability. It was reported [6,8] that both medium- and high-Mn steels may show some manufacturing problems during forming related to the their plastic instability.

The explanation of the Portevin–Le Chatelier mechanism in medium-Mn steels showing the TRIP effect is a complicated issue because of their microstructure consisting of several phases, as well as the TRIP effect exhibited by these steels. The exact characteristics of the factors affecting the PLC effect in AHSS is very important, both from a research point of view and their industrial implementation. This overview concerns the PLC phenomenon in Advanced High Strength Steels (AHSSs), with particular emphasis on advanced medium-Mn TRIP steels.

## 2. The Nature of PLC Effect in Steels

The plastic instability phenomenon occurring during the deformation of metallic materials shows two most common forms of propagative bands: Lüders and Portevin–Le Chatelier bands. The Lüders bands refer to the regions of localized strain. They form immediately after the onset of plastic deformation from the yield point drop, followed to a dominant stage of the stress plateau stage. Lüders bands are commonly caused by static strain aging (SSA) [13]. Static strain aging is characterized by an increase in strength properties associated with a decrease in plasticity. The PLC bands are represented by characteristic serrations on stress–strain curves. PLC bands are usually related to the dynamic strain aging (DSA) effect. The occurrence of the PLC bands is much more erratic, and can be observed in various forms (serration types) in comparison to the Lüders bands.

There are several theories which explain the PLC effect in metallic materials. However, none has been so far clearly confirmed. The first interpretation of this phenomenon was proposed by Cottrell [1]. From his point of view, the PLC effect is related to the interactions between solute atoms, such as C or N, and mobile dislocations. The presence of serrations on a tensile curve is associated with the rapid release of dislocations from the atmospheres of dissolved atoms, which block their movement. This model is based on the assumption that atmospheres are formed around dislocations due to volume diffusion. In the presence of substitution atoms, diffusion is facilitated by vacancies resulting from plastic deformation. The interstitial gaps located in the vicinity of the dislocation are enlarged due to a plastic deformation of the crystal lattice, so substitution atoms get into such enlarged gaps, creating the Cottrell atmospheres. To form such atmospheres, the diffusion rate of the dissolved atoms must be slightly lower than the dislocation movement. Rapid unpinning of dislocations from atmospheres is accompanied by a drop on a stress–strain curve, because the dislocations released from obstacles can move. If the pinning and unpinning processes are repeated cyclically, it leads to a DSA effect [13]. At a sufficiently high temperature the diffusion rate is high enough, therefore solute atoms can move at the same rate as the dislocations, whereas at sufficiently high deformation rates, or under conditions of low diffusion intensity, Cottrell atmospheres at dislocations are not formed [1]. McCormic [14] completed the Cottrell theory with a statement, that mobile dislocations do not move uniformly; they are stopped temporarily at obstacles during plastic deformation and then the formation of Cottrell atmospheres takes place. Penning [15] reported that homogeneous plastic deformation is unstable when stress decreases as well as the strain rate increases. This effect is called negative strain rate sensitivity (NSRS). The main factors causing the NSRS are the DSA suppression by an increment in dislocations velocity and the inhibition of deformation twinning under high strain rates [8,9].

The heterogeneous plastic deformation phenomenon is usually preceded by a homogeneous deformation. A strain level at which the serrations appear is referred to a critical strain initiating the PLC effect. The value of critical strain is related to several factors including solute atom concentration, dislocation density, grain size, deformation temperature and strain rate [3,4,8,9]. Equation (1) includes most of these factors. Moreover, the activation energy for serration occurrence can be estimated [8]:

(1) $$\dot{\varepsilon }=KC^{n}\exp{\left(-\frac{Q_{M}}{RT}\right)}_{c}^{m}$$

$\dot{\varepsilon }$ is strain rate, *K*, *m* and *n* are constants, *C* is the carbon content, *Q_M_* is the activation energy for serrations occurrence, *R* is gas constant, *T* is the deformation temperature, *ε_c_* is a critical strain value at which the serrations appear.

**Figure 1:**
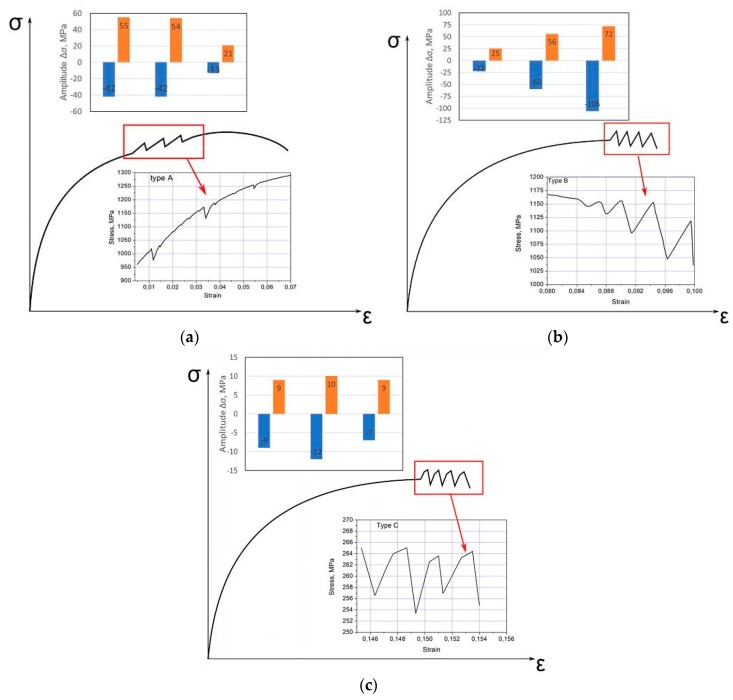
Types of oscillations and examples of the histograms of the serration flow: (**a**) type A, (**b**) type B and (**c**) type C.

Types of serrations were classified by Brindley and Worington [16] as A, B or C. The type A usually appears at ambient deformation temperature. It is characterized by a rise and then a rapid fall in stress value, while oscillations repeat periodically. The A-type serrations are irregular, with relatively small stress drops on tensile curves. The histogram in Figure 1a presents the distribution of stress drops; it shows that the amplitude of serrations is relatively low. The B-type occurs at elevated deformation temperatures. It is usually preceded by the presence of A-type oscillations. Oscillations of B type appear regularly on a σ–ε curve. The amplitude of oscillations is higher when compared to A-type serrations (Figure 1b). Serrations of C-type are related to oscillations, whose maximum values do not exceed an average level of the tensile curve. The amplitude of oscillations observed in such kinds of serrations is more regular when compared to serrations of A- or B-types (Figure 1c).

Literature studies have shown that the PLC effect occurs not only in copper, aluminum and nickel alloys [2,3,4,17,18], but also in some steel grades. Plastic instability phenomenon was observed in steels with increased Cr content [7,19], austenitic stainless steels [20], Hadfield steels [21] and dual phase (DP) steels [22]. Some of the AHSSs are also prone to plastic instability phenomenon, such as high-manganese Twinning-Induced Plasticity (TWIP) steels [8], medium-manganese steels [6,13] and Q&P steels [10].

## 3. Theories Explaining the PLC Mechanism in Medium- and High-Manganese Steels

Several theories explaining the PLC mechanism in high-Mn (17–30% wt % Mn) and medium-manganese (3–12% wt % Mn) steels are available in the literature. The DSA mechanism in these steels is related to the interaction between interstitial and substitutional atoms and the stacking fault energy (SFE). With an increase in the SFE (for example by rising temperature), the DSA effect contributes to the work hardening of high-manganese steels containing a high C concentration [8]. An increase in the manganese content in steel results in a reduction in the rate of carbon diffusion. This is the reason why the PLC effect is so pronounced in the high-Mn steels (Table 1). The DSA occurrence at room temperature is difficult in austenitic steels due to their low carbon diffusivity. Therefore, Owen and Grujicic [21] suggested that the DSA effect in steels with increased Mn content is caused by interaction between dislocations and manganese–carbon clusters which enable the occurrence of DSA even at room temperature. Changing the position of C atoms between octahedral and tetrahedral interstices within clusters may lock partial dislocations in stacking faults. The octahedral position is energetically stable for C. During plastic deformation, carbon changes position to a tetrahedral, which results in the generation of a stacking fault. The location of carbon in the tetrahedral site is not stable, and thus it returns to an octahedral position. When the carbon moves back to the same position in the octahedral site, a dislocation pinning effect associated with the DSA does not occur. If a C atom jumps to the position where a Mn atom is located, the amount of C–Mn pairs in the stacking faults rises, which prevents dislocations motion resulting in the occurrence of DSA [8]. Hickel et al. [23] proved a strong impact of Mn–C clusters on the SFE. They showed that the active deformation mechanism redistributes local solutes, and thus significantly affects the change in the local SFE. However, Medvedeva et al. [24] reported that the presence of the Mn–C pairs limits the forecasted impact in the SFE that is caused by carbon. Kang et al. [25] suggested that short range reorientation C–Mn clusters could be sheared by moving dislocations and leads to glide plane softening, resulting in an increase of the glide of the planar and a stress plateau in a flow curve.

Factors influencing the DSA effect in medium- and high-manganese steels are also related to the amount of Al and Si additions and grain size. Reducing the interaction time between dislocations and Mn–C complexes explains the reduction of the DSA effect. Al delays the reorientation of Mn–C point defect complexes by rising the process activation energy [28,31,32,33,34]. Song et al. [35] and Madivala et al. [36] reported that some amount of Al addition could suppress the Mn–C formation in high-Mn steels. This might be the reason for the delay or absence of the serration phenomenon in Al-alloyed high-Mn steels. Si addition also causes the increase in critical strain for serrated flow [8,31,33]. In high-manganese austenitic steels the influence of grain size on the plastic instability phenomenon is different than in other steels. Typically, the DSA effect is enhanced in fine-grained steels. However, in case of high-Mn austenitic steels, a critical strain for serrated flow rises with the reduction of grain size. It is related to the fact that dislocation density in early stage of deformation is lower for smaller austenite grains [37,38]. Presence of carbides or complex carbonitrides also reduces the probability of DSA. It was reported [39] that an addition of 4.6 wt % Cr to the medium-Mn 0.17C–12Mn–4.5Cr–1.2Si steel significantly reduced the DSA by precipitation of M_23_(C,N)_6_ occurring during batch annealing (600 °C) within 20 h. It is related to a reduction in the carbon content in the solid solution.

### 3.1. Effect of Twinning-Induced Plasticity (TWIP) on Plastic Instability Phenomenon

In the group of AHSSs, the PLC effect is the most characterized in case of high-Mn TWIP steels containing 17–30% Mn [25,26,27,28,29,31,32,33,34]. In these steels, work hardening is related to the PLC and TWIP effects which occur simultaneously. Some authors reported that deformation twinning is a very important mechanism responsible for the heterogeneous deformation in high-manganese TWIP steels [25,28,31]. Allain et al. [37] suggested that dynamic interactions between carbon atoms and dislocations could suppress dislocation glide due to the lattice friction effects, and as a result promote deformation twinning. Lebedkina et al. [28] observed that serrations on tensile curves have an influence on the activation of twinning systems. Sevsek et al. [31] reported that inhibiting the movement of partial dislocations by ordered C–Mn short-range clusters promotes twin deformations. The deformation twinning activates additional sources of displacement, sliding systems and twin systems in nearby grains. At the macroscopic level, this generates local deformation phenomena such as deformation bands. They are detectable in the form of a serrated flow in steels containing high-Mn contents. They also noticed that the occurrence of serrated flow is dominated by the deformation temperature (150 °C). This promotes C diffusivity, thereby improving the twin deformation in a short range. They also found the correlation between the occurrence of serrated flow behavior and material state. In the case of microstructures with the high homogeneity, for example a crystallized structure, localized activation of new deformation mechanisms strengthened the creation and propagation of deformation bands and the serrated flow in high-Mn TWIP steels. In the case of structures without homogeneity, for example, partially-recrystallized, this process is limited. This is due to the inhibition of the propagation of deformation bands.

### 3.2. Effect of Transformation-Induced Plasticity (TRIP) on Plastic Instability Phenomenon

So far, the PLC effect in medium-manganese TRIP steels has not been characterized in detail. Only a few publications concerning plastic instability phenomenon in these steels are available in the literature [6,13,40,41,42,43,44]. The explanation of the DSA mechanism in medium-manganese steels showing the Transformation-Induced-Plasticity effect is a complex issue because of their microstructure consisting of several phases, as well as the TRIP effect exhibited by these steels. There are a few theories concerning the nature of plastic instability in medium manganese steels. Gibbs et al. [45] proposed an explanation about the characteristic serrations with different rates of martensitic transformation during deformation. Sun et al. [41] pointed to the association of the plastic instability of 0.26C–11.6Mn–2.7Al and intermittent martensitic transformation-induced by strain. They noticed localized martensite transformation occurring in the PLC areas, which spread when they are deformed. Callahan et al. [40] noticed in the 0.2C–5Mn–2.5Al steel at the same time a TRIP effect activity and the presence of both Lüders and PLC bands.

Ryu et al. [42] found that the localized strain triggered by Lüders band may cause the austenite transformation into martensite. PLC bands are a form of strain localization, in which the metastable austenite grains have good conditions to martensitic transformation because of the severe strain [6,42,44]. However, strain-induced martensite acts as obstacles for propagation of PLC bands. Yang et al. [6] reported that PLC bands observed in 0.3C–7Mn–2Al steel were subjected to a dynamic hindrance caused by the inside-band transformed martensite during propagation. For this reason a volume fraction of the dynamically formed martensite should affect the characteristics of Portevin–Le Chatelier bands.

**Figure 2:**
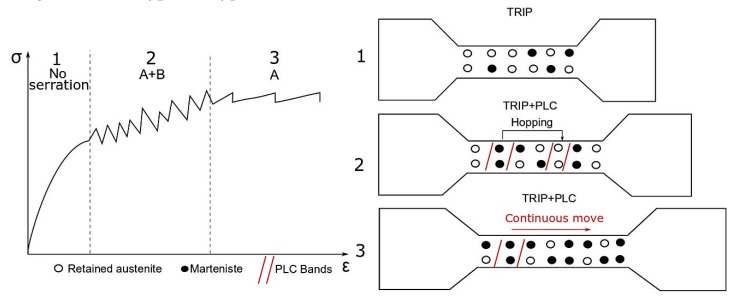
The scheme showing the relationship between a serration type and stability of retained austenite in Transformation-Induced Plasticity (TRIP) steels deformed at room temperature.

Figure 2 explains the effect of strain-induced martensitic transformation on the serrations characteristics. The retained austenite gradually transforms into martensite during straining, and the amount of newly formed martensite increases as the deformation level increases. Austenite easily transforms into martensite in areas of localized strain, such as PLC bands. This microstructural component is characterized by higher hardness than austenite. Hence, it acts as obstacles for PLC bands propagation. The presence of martensite affects a type of oscillations observed on tensile curves. At the initial deformation level, a significant amount of retained austenite transforms into martensite. Thus, high stress is needed to continue the propagation of PLC bands. Under such conditions, the new area becomes a source of PLC bands, enabling them to move under relatively low stresses. This mechanism is a reason of PLC bands hopping. If the retained austenite is characterized by relatively low stability, it easily transforms into martensite resulting in occurring irregular serrations characterized by a various amplitude (type A+B or B). In the late stage of deformation, characterized by the presence of the only most stable non-transformed grains of retained austenite and uniformly-distributed obstacles (martensite areas), the propagation of PLC bands is more regular. That is why PLC bands move continuously. As a result, oscillations of the A-type characterized by a lower amplitude than B-type can be observed on tensile curves. The serrations changed from A+B type to A type in this case.

## 4. The PLC Effect in Medium-Manganese Steels

In general, plastic instability phenomenon in medium-manganese steels is related to a DSA effect. The mechanism of the Portevin–Le Chatelier effect in these steels is very similar to that in high-manganese steels due to increased Mn content. Due to a lower Mn content (5–12%), medium-Mn steels are less prone to an occurrence of plastic instability phenomenon [46]. It was found [30] that an increase in a Mn content from 3% to 5% in C–Mn–Al medium-manganese steels results in the appearance of the PLC effect. It is related to higher amount of C–Mn pairs which interact dynamically with dislocations during deformation.

The occurrence of plastic instability phenomenon in medium-Mn steels is also related to a grain size. It was reported [6,39] that the dislocation density in austenite is influenced, not only by the level of deformation, but also by the size of the austenite grain. If the austenite grain is less than the critical value, sufficient dislocations cannot be generated during deformation. The dislocation density increased more slowly in fine-grained austenite, so the amount of dislocations cannot be enough to induce the PLC band formation.

### 4.1. Cold-Rolled Medium-Mn Steels

**Figure 3:**
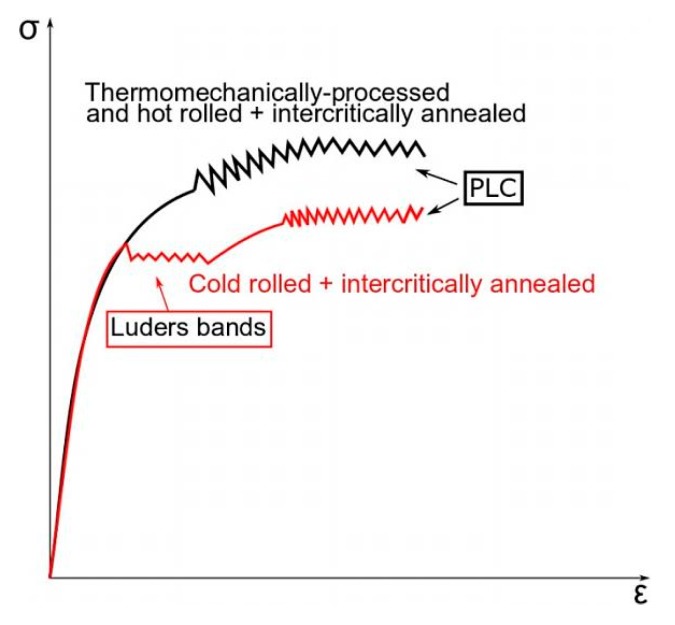
Types of plastic instability in medium-manganese steels.

Most reports concerning the Portevin–Le Chatelier effect in medium-manganese steels are related to cold-rolled grades characterized by ferritic-austenitic microstructure [6,13,40]. Steels obtained by cold rolling are subjected to intercritical annealing. They are also prone to Lüders band formation (Figure 3). Values of the Lüders strain depend on a variety of factors such as chemical composition, strain rate, grain size and deformation temperature, similarly to the formation of PLC bands. However, PLC bands observed as serrations on tensile curves appear less regularly when compared to Lüders bands. The presence of Lüders and PLC bands was observed by Wang et al. [13] and Sevsek [47] in cold-rolled and intercritically-annealed 7Mn–0.14C–0.23Si and X6MnAl12-3 steels, respectively. Callahan et al. [40] reported that the temperature of intercritical annealing affects the occurrence of PLC and Lüders bands. Serrations observed in 0.2C–5Mn–2.5Al steel disappeared when the temperature of intercritical annealing was rising from 740 to 780 °C. Similar results were reported by Yang et al. [6]. They also observed a serrated flow in 0.22C–7.2Mn–2.4Al steel only at annealing temperatures 700 °C and 720 °C. In intercritically-annealed steels, PLC bands can nucleate only in the austenite grains because of their higher carbon content than in ferrite [6].

### 4.2. Hot-Rolled Medium-Mn Steels

The PLC effect in medium-manganese steels manufactured by cold rolling has sparsely been investigated [30,46,48]. In contrast to cold-rolled medium-Mn steels, hot-rolled grades do not show a clear yield point as well as the next Lüders elongation (Figure 3). The continuous yielding behavior is the result of mobile dislocations occurring during thermomechanical rolling [30,46,48]. The disappearance of the PLC effect in such steels can be also related to cementite precipitation which reduces a carbon content in solid solution, so the dynamic interaction with dislocations can be eliminated [48]. However, some processing factors could trigger the PLC effect in hot-rolled medium-manganese steels. It was reported [42] that the time and temperature of isothermal holding in an intercritical region affect the appearance of the plastic instability effect.

## 5. Effect of Deformation Temperature on the PLC Effect

An effect of elevated temperatures on the PLC related to dynamic strain aging (DSA) in medium- and high-manganese steels have received minimal attention in the literature. The plastic deformation temperature has a huge impact on the mechanical properties obtained during the production of steel elements. Min et al. [10] reported that strain rates in automotive stamping processes can reach 1–10 s^−1^ with minimal heat dissipation. During the stamping process a temperature of even ~280 °C can be generated. However, during typical production operations, this temperature does not exceed ~130 °C [10,49].

**Figure 4:**
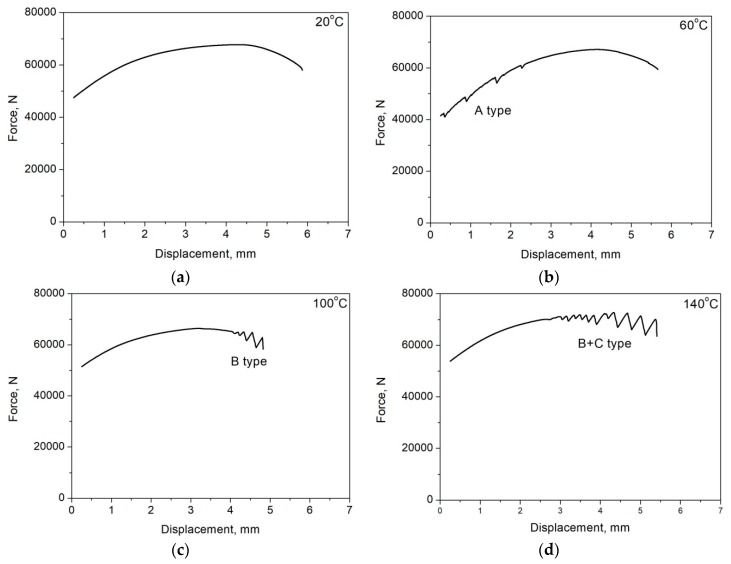

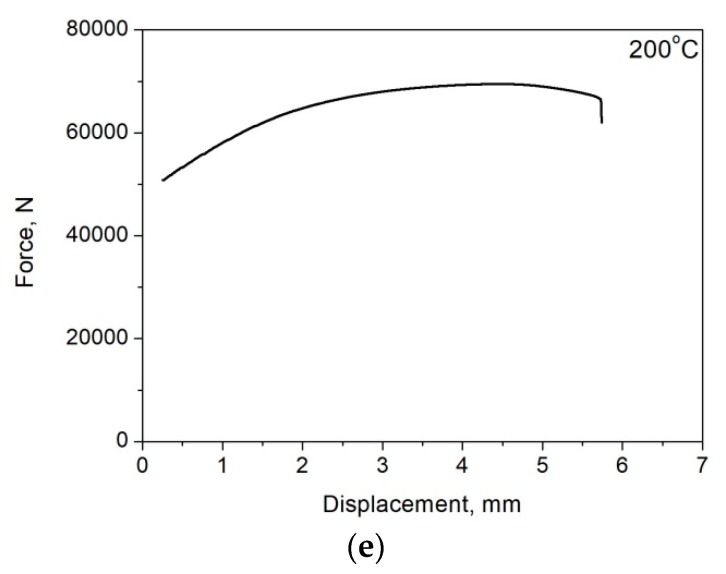
Tensile curves of the investigated hot-rolled 0.16C–4.7Mn–1.6Al–0.22Si–0.20Mo steel registered at: (**a**) 20 °C, (**b**) 60 °C, (**c**) 100 °C, (**d**) 140 °C and (**e**) 200 °C.

The DSA occurs in a specific range of temperature, and a value of critical strain activating a PLC effect is related to the deformation temperature (Table 1). Min et al. [10] reported that a value of critical strain in 0.2C–2Mn–1.4Si QP steel increases with decreasing temperature. However, in works [30,46] concerning the plastic instability phenomenon in medium-Mn steels, the opposite trend was observed. Grzegorczyk et al. [30] reported that a value of critical strain in 0.16C–4.7Mn–1.6Al–0.22Si-0.20Mo steel is higher at deformation temperatures of 100 and 140 °C than at 60 °C (Figure 3). The temperature drop in steels with a higher manganese content causes longer reorientation time of the C–Mn complex, which suppresses the DSA. It can be seen in Figure 4 that serrations occur at the deformation temperature range of 60–140 °C, whereas they were not observed in specimens deformed at 20 and 200 °C. Min et al. [10] observed the similar tendency in steel deformed at a temperature range of 25–350 °C. Serrations were not observed at deformation temperatures below 100 °C and higher than 250 °C. They concluded that at low temperatures where serrations do not occur, dislocation pinning occurs very slow compared to dislocation unpinning. At high temperatures, where serrations do not occur, a solute diffusion rate is high enough to reduce the pinning force on dislocations.

The disappearance of the PLC effect at high deformation temperatures can be also related to carbide precipitation. Kipelova et al. [7] investigated the effect of deformation temperature in a range 200–500 °C in 0.13C–8.6Cr–3.2Co–1.2W–0.9Mo steel. They found that at the deformation temperature higher than 350 °C, serrations disappeared due to carbide precipitation, which resulted in a reduced concentration of interstitial atoms in solid solution, terminating the DSA. The decreasing carbon content due to carbide precipitation requires a larger strain to trigger the Portevin–Le Chatelier effect. The very similar situation took place in the investigated 0.16C–4.7Mn–1.6Al–0.22Si–0.20Mo steel (Figure 4). Moreover, at deformation temperatures 100 and 140 °C, serrations were observed in a post-uniform elongation range. Kipelova et al. [7] observed a similar effect in a temperature range of 200–300 °C. This was explained because of the carbide precipitation effect, which increased the critical deformation activating the DSA to a value greater than the uniform deformation of the steel. Therefore, no PLC effect could be observed prior to necking.

Jung and De Cooman [31] investigated the effect of deformation temperature in 0.6C–18Mn–2.5Al steel. They observed the relation between deformation temperature and a type of serrations. The B-type was occurred at the largest deformations at high temperatures. It was related to the fact that the increase in temperature increases the carbon diffusion. Hence, carbon atoms did not form C–Mn clusters, and the diffusion path shortens as the strain increases because of a high dislocation density. The combination of these effects can lead to a rapid uptake of dislocation by C atoms leading to the observed B-type serration. The same type of serrations show the specimens deformed at 100 and 140 °C (Figure 4).

**Figure 5:**
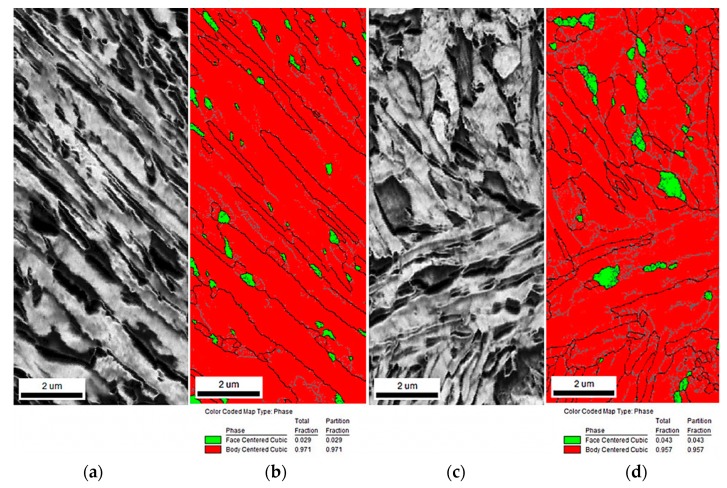
Image quality (IQ) maps of the 0.17C–3.1Mn–1.6Al–0.22Si–0.22Mo steel deformed at: 20 °C (**a**), 140 °C (**c**) and phase distribution maps obtained at: 20 °C (**b**), 140 °C (**d**).

Deformation temperature affects the stability of retained austenite (RA). It is well documented in the literature [30] that stability of retained austenite increases at higher deformation temperatures. Very helpful in identifying strain-induced martensitic transformation is an Electron Backscatter Diffraction (EBSD) method. Figure 5 shows the image quality (IQ) and phase distribution maps. Martensite is characterized by the highest crystal lattice distortion. Thus, it occurs in the darkest areas, showing the lowest IQ parameter (Figure 5a,c). The retained austenite is located at the dark areas, whereas bainitic ferrite is characterized by a slightly higher IQ value. Obtained results show that the specimen deformed at the higher temperature 140 °C (Figure 5c) possesses a smaller fraction of the darkest areas when compared to the specimen deformed at (the lower) 20 °C (Figure 5a). It is related to the fact that austenite is more stable at the higher deformation temperature. The EBSD method allows also the amount and morphological details of retained austenite to be determined (as green in Figure 5b,d). RA easier transforms into martensite at the lower deformation temperature (Figure 5b).

## 6. Effect of Strain Rate on the PLC Effect

The effect of strain rate in medium- and high-Mn steels is usually discussed in terms of negative strain rate sensitivity (NSRS), which reduces the amount of work hardening at high strain rates [47,51,52,53]. Usually, conventional TRIP steels do not show this effect [48]. The occurrence of the DSA effect can be reduced by a significant increase of strain rates during plastic deformation. Several authors reported that the NSRS effect is related to the stability of RA due to adiabatic heating at high strain rates. Generally, adiabatic heating affects both the DSA and NSRS by increased diffusion intensity [8,9,15,49]. Callahan et al. [52] reported that the NSRS in 0.2C–5Mn–2.5Al TRIP steel is due to the effect of adiabatic heating, which increases the stability of retained austenite, and thus reduces the work hardening rate. They found that the NSRS in medium-Mn TRIP steel does not follow from the DSA. As the martensitic transformation rate increases, the smallest deformation rate required to generate uniform deformation decreases, similarly to fall in a deformation temperature. This was explained by the formation of dislocations that could move during martensitic transformation. Seol et al. reported [26] that the high strain rate deformation suppresses the DSA in 0.2C–17Mn steel; thus a ductility increase and lower yield strength were noted. However, an increase in a strain rate results also in an increased stability of the retained austenite due to adiabatic heating. Despite this, these effects are balanced, so increased ductility was noted.

Based on the results performed on non-ferrous alloys, it is well known that the PLC effect is also strain rate-dependent (Table 1) [4]. The increase in strain rate reduces interaction time between dislocations and carbon atoms or C–Mn clusters. Hence, it can significantly reduce the PLC effect [6]. When the strain rate decreases, the waiting time of the dislocations rises, and the magnitude of the serrations is enhanced [50]. Min et al. [10] reported that a value of critical strain rises with an increasing strain rate in QP steels.

## 7. Conclusions

The Portevin–Le Chatelier (PLC) phenomenon in AHSSs was analyzed. The PLC effect in medium- and high-Mn steels was discussed based on the theory concerning the interaction between dislocations and Mn–C clusters. A mechanism of the Portevin–Le Chatelier effect in these steels is similar due to Mn alloying. The PLC is affected by microstructural mechanisms such as Twinning-Induced Plasticity (TWIP) and Transformation-Induced Plasticity (TRIP) effects. The appearance and intensity of plastic instability phenomenon are related to several factors. In this study, a special attention was paid to deformation temperature and strain rate, which occur during processing of steel sheets. In order to improve the formability of automotive medium-Mn steel sheets, the PLC effect should be suppressed. The occurrence of plastic instability in these steels can be reduced by:

- Reduction of carbon and manganese contents. It results in a smaller amount of C–Mn pairs, which interact dynamically with dislocations during deformation.
- Addition of elements which form carbides. Carbide precipitation decreases the concentration of interstitial atoms in solid solution, terminating the DSA. The lower carbon content due to carbide precipitation requires a larger strain to trigger the PLC effect.
- Reduction in grain size. The dislocation density increases more slowly in fine-grained austenite grains. Hence, the amount of dislocations cannot be high enough to induce the PLC band formation.
- Application of hot rolling rather than cold rolling. Hot-rolled steel grades are not prone to Lüders band formation due to the presence of mobile dislocations generated during thermomechanical processing.
- Increasing the strain rate during deformation. It reduces interaction time between dislocations and carbon atoms C–Mn clusters. Thus, it can significantly reduce the PLC effect. However, applying a high deformation rate results in the higher stability of retained austenite because of adiabatic heating, which stimulates the lower intensity of the TRIP effect.

## Figures and Tables

**Table 1:** The value of critical serration strain in steels with various Mn content deformed at different strain rate and temperature.

Type of Steel.	Critical Strain, %	Deformation Temperature, °C	Strain Rate s^−1^	Microstructure	Reference
0.18C–17Mn	~12	room temperature	10^−4^	austenite+martensite	[26]
0.18C–17Mn	~15	room temperature	10^−3^	austenite+martensite	[26]
0.18C–17Mn	~16	room temperature	10^−2^	austenite+martensite	[26]
0.6C–22Mn	~5	room temperature	10^−3^	austenite	[27]
0.6C–18Mn	~15	room temperature	10^−3^	austenite	[27]
0.6C–18Mn–2Al	no serrations	room temperature	10^−3^	austenite	[27]
0.3C–17Mn–1Al	25	150	25 × 10^−3^	austenite	[28]
0.6C–18Mn	5	150	25 × 10^−3^	austenite	[28]
0.6–18Mn	3	room temperature	25 × 10^−3^	austenite	[28]
0.2C–2Mn–1.4Si	~5	100	5 × 10^−5^	ferrite+martensite+ retained austenite	[10]
0.2C–2Mn–1.4Si	no serrations	200	5 × 10^−5^	ferrite+martensite+ retained austenite	[10]
0.3C–7Mn–2Al	~10	−50	6.67 × 10^−4^	ferrite+austenite	[7]
0.3C–7Mn–2Al	~25	27	6.67 × 10^−4^	ferrite+austenite	[7]
0.3C–10Mn–3Al–2Si	43	room temperature	10^−3^	ferrite+austenite	[29]
0.3C–9Mn–2Al	~10	room temperature	10^−3^	ferrite+austenite	[29]
0.16C–5Mn–1.6Al	~1	60	10^−3^	bainite+retained austenite	[30]
0.16C–5Mn–1.6Al	~8	100	10^-3^	bainite+retained austenite	[30]
0.16C–5Mn–1.6Al	~6	140	10^-3^	bainite+retained austenite	[30]
